# Is cardiorespiratory fitness associated with cognitive outcomes in mid‐adulthood? Findings from the 1958 British birth cohort

**DOI:** 10.1111/sms.14525

**Published:** 2023-10-18

**Authors:** S. M. Pinto Pereira, J. J. Mitchell, J. M. Blodgett, M. Hamer, T. Norris

**Affiliations:** ^1^ Institute of Sport, Exercise and Health, Division of Surgery and Interventional Science, Faculty of Medical Sciences UCL London UK

**Keywords:** cardiorespiratory fitness, cognition, cohort, epidemiology

## Abstract

Identifying causal factors to intervene on to delay age‐related declines in cognitive function is urgently needed. We examined associations between non‐exercise testing cardiorespiratory fitness (NETCRF; estimated using sex, age, body mass index, resting heart rate, and physical activity) at 45 years and cognitive function outcomes (immediate and delayed verbal memory; verbal fluency; visual processing speed) at 50 years in 8130 participants from the 1958 British birth cohort. In unadjusted models, higher NETCRF was associated with better cognitive function across all outcomes. When adjusted for confounding factors, associations disappeared. In this cohort, associations between 45 years NETCRF and 50 years cognitive function likely result from confounding factors.

## 1 BACKGROUND

Cognitive impairment in later life is a public health concern as it is associated with risk of progression to various dementias.^1^ Dementia is a major cause of disability among older people, accounting for 11.9% of years lived with disability due to noncommunicable diseases.^2^ In light of population aging, strategies to alleviate cognitive impairment (and therefore dementia), will be even more relevant in the coming decades.^2^ There are no effective treatments in Europe to reverse or delay dementia progression. Therefore, identifying causal factors which can be intervened upon to delay age‐related declines in cognitive function, is of urgent need.

Cardiorespiratory fitness (CRF) is a marker of the capacity of the cardiovascular system to transport oxygen and the ability of muscle tissue to utilize it.^3^ It is influenced by numerous processes (e.g., mechanical ventilation, external and internal respiration, ventricular contraction, cellular vascularization), occurring across several systems (e.g., respiratory, cardiovascular, neuromuscular, metabolic). In light of strong evidence for an inverse relationship between CRF and risk of cardiovascular disease, cancer and all‐cause mortality, the American Heart Association (AHA), proposed that CRF should be used as a clinical vital sign.^3^

There is less evidence regarding the association between CRF and cognitive function in later life. While some evidence suggests a predictive effect of CRF in adulthood on cognitive function,^4^ recent reviews noted that most studies investigating the CRF―cognitive function association were cross‐sectional.^5^ While cross‐sectional studies can help identify potential relationships, longitudinal studies are needed to establish causality between CRF and cognitive function. If a causal relationship exists between CRF and cognitive function in later life, such knowledge can contribute to developing intervention trials and policy recommendations. In so doing, this would ensure that observed increases in longevity are characterized by healthy cognitive aging.

Here, we utilized longitudinal data from the 1958 British birth cohort study^6^ to investigate whether CRF at 45 years was associated with cognitive function at 50 years.

## 2 METHODS

Data come from the 1958 British birth cohort study.^6^ The study enrolled 17 638 participants at birth during a single week in March 1958 in Scotland, Wales and England and a further 920 immigrants born in the same week. In mid‐adulthood (45 years), it remains broadly representative of the original study sample.^7^ At 45 years (2002/2003; *N* = 9377) and 50 years (2008; *N* = 9790), study nurses visited participants in their homes and obtained biomedical (45 years only), physical and sociodemographic characteristics.

Our analytic sample (*N* = 8130) includes participants with at least one valid measure of cognitive function at 50 years and who attended 45 years sweep when CRF was derived (see Figure S1).

### 2.1 ETHICS STATEMENT

At both waves, informed consent was obtained from all participants. Ethical approval was given by the South‐East multi‐centre research ethics committee (01/1/44) for age 45 years sweep and by the London multi‐centre research ethics committee (08/H0718/29) for the 50 years sweep.

*Exposure*: Non‐exercise testing cardiorespiratory fitness (NETCRF, 45 years).

NETCRF was predicted using information on sex, age, body mass index (BMI), resting heart rate (RHR), and self‐reported physical activity (PA). Such prediction equations have demonstrated strong validity (*r*: 0.76–0.81) against exercise testing‐estimated fitness.^8^ At 45 years, weight and height were measured by trained nurses using standard protocols; BMI (kg/m^2^) was calculated. Three measurements of resting heart rate were obtained (taken 1 min apart) after a few minutes rest using an automated device (Omron 907 blood pressure monitor, Omron Healthcare, Milton Keynes, UK); the mean was calculated. Age was recorded in whole years. A modified version of the EPIC Physical Activity Questionnaire (EPIC‐PAQ) was used to assess leisure‐time PA.^9^

NETCRF was calculated according to the equation developed by Jurca et al (2005) using data from a UK population^8^:

$$\text{NETCRF}\;\left(\mathrm{mL}/\mathrm{kg}/\min\right)=\left[\mathrm{sex}\;\text{coefficient}\times 2.78-\left(\mathrm{age}\times 0.11\right)-\left(\mathrm{BMI}\times 0.17\right)-\left(\mathrm{RHR}\times 0.05\right)+\left(\mathrm{PA}\;\text{level coefficient}\right)+21.41\right].$$

The sex coefficient was one for males and zero for females. The PA coefficient was based on adherence to contemporaneous guidelines assessed using the EPIC‐PAQ^9^: 0.0 for inactive during leisure‐time; 0.29 for active, but not meeting guidelines; and, 1.21 for meeting guidelines of at least 150 min/week of moderate‐intensity or 75 min/week of vigorous‐intensity PA. NETCRF estimates were converted into maximal aerobic capacity metabolic equivalent (MET) values (1 MET corresponds to oxygen consumption of 3.5 mL/kg/min (based on a 70 kg male aged 40 years)).

*Outcome*: Cognitive function (50 years).

Four indicators of cognitive function were measured, completed in the following order: (i) immediate verbal memory; (ii) verbal fluency; (iii) visual processing speed; and (iv) delayed verbal memory. For immediate verbal memory, participants were played an audio recording of 10 words and were then given 2 min to orally recall them. Verbal fluency was assessed via an animal naming test, in which respondents were given 1 min to name as many animals as possible. Visual processing speed was assessed using a dual‐letter cancellation test, in which participants were presented with blocks of letters and were asked to read through the blocks from left to right, crossing out “Ws” and “Ps” as they read. Search speed was calculated by summing the total number of letters scanned, including both target and non‐target letters. Delayed memory was tested by asking participants to recall as many words as they could from the original list presented to them during the first word‐recall task, with a two‐minute cut‐off. The four cognitive function outcomes were standardized (z‐score) to address differences in the units of measurement. The average of the four z‐scores was calculated and represented an overall composite cognitive function score.

### 2.2 Putative confounders

Potential confounders of the NETCRF―cognitive function association were identified a‐priori and included in the directed acyclic graph (Figure S2). They included: social class at birth, childhood cognitive function (11 years), educational attainment (33 years), PA level (42 years), smoking status (42 years), alcohol consumption (42 years), BMI (42 years) and self‐rated health over the previous 12 months (42 years). See Supplementary material for further details.

### 2.3 Statistical analysis

We used linear regression to model the relationship between NETCRF at 45 years and each of the five cognitive function outcomes. As the cognitive measures were standardized, the regression models estimate the effect of a 1 MET change in NETCRF on cognitive outcome standard deviation (SD) scores. We fitted unadjusted models and then adjusted for the putative confounders listed above. Due to observed sex‐differences in the relationship between CRF and a number of outcomes, including brain function,^10^ an a‐priori decision was made to perform sex‐stratified analyses.

To address missingness in covariate data (see Table 1), we used multiple imputation by chained equations, under a missing‐at‐random assumption. All variables described above (i.e., NETCRF at 45 years, cognitive function outcomes at 50 years and putative confounding variables) were included in the imputation model, as well as childhood internalizing and externalizing behaviors which have been used previously in this cohort to predict missingness in follow‐up.^7^

**TABLE 1:** Sample characteristics^a^.

Variable (reporting age (years))	Missing (*n* (%))	Males	Females
Sex (birth)	‐	3992 (49.1)	4138 (50.9)
NETCRF (45 years)	2150 (26.4)		
(METS)		11.8 (1.2)	9.0 (1.3)
kg/ml/min		41.4 (16.7)	31.7 (4.6)
Weight (kg) (45 years)	111 (1.4)	85 (76.4, 94.4)	68.2 (60.8, 78.4)
Height (cm) (45 years)	78 (1.0)	176.1 (6.7)	162.6 (6.2)
BMI (kg/m^2^) (45 years)	140 (1.7)	27.3 (25.0, 30.1)	25.7 (23.1, 29.7)
Resting heart rate (45 years)	64 (0.8)	70.4 (11.1)	72.5 (10.0)
Immediate verbal memory (Number of words correctly recalled) (50 years)	‐	6.4 (1.5)	6.7 (1.5)
Verbal fluency (Number of animals mentioned) (50 years)	‐	22.4 (6.4)	22.4 (6.1)
Visual processing speed (Total number of letters scanned) (50 years)	149 (1.8)	312 (270, 372)	325 (284, 400)
Delayed verbal memory (Number of words correctly recalled after delay) (50 years)	53 (0.7)	5.2 (1.8)	5.7 (1.8)
Social class (birth^b^)	‐		
Professional/managerial		799 (20.0)	794 (19.2)
Skilled non‐manual		405 (10.2)	400 (9.7)
Skilled manual		1888 (47.3)	1984 (48.0)
Semiskilled/unskilled manual/no male head		900 (22.6)	960 (23.2)
Childhood cognitive function (mean reading and maths score) (11 years)	1037 (12.8)	17.8 (7.7)	17.5 (7.2)
Educational attainment (33 years)	917 (11.3)		
No qualifications		492 (14.1)	446 (12.0)
CSE/O‐level		1992 (57.2)	2256 (60.5)
A‐level		376 (10.8)	454 (12.2)
>A‐level		621 (17.8)	576 (15.4)
Smoking status (42 years)	201 (2.5)		
Never		1766 (45.5)	1944 (48.0)
Ex‐smoker		1065 (27.5)	1001 (24.7)
Current		1049 (27.0)	1104 (27.3)
Alcohol consumption frequency (42 years)	200 (2.5)		
Never		134 (3.5)	228 (5.6)
Rarely		305 (7.9)	668 (16.5)
Two to four times per month		1058 (27.3)	1311 (32.4)
At least twice per week		2384 (61.4)	1842 (45.5)
BMI (kg/m^2^) (42 years)	422 (5.2)	25.9 (23.8, 28.4)	24.0 (21.9, 27.3)
Physical activity (42 years)	202 (2.5)		
≤3 per month		1370 (33.8)	1289 (33.2)
Once per week		699 (17.3)	814 (21.0)
Two to three times per week		867 (21.4)	854 (22.0)
Four to seven times per week		1112 (27.5)	923 (23.8)
Self‐rated health over previous 12 months (42 years)	199 (2.5)		
Excellent		1171 (30.2)	1111 (27.4)
Good		1848 (47.6)	1907 (47.1)
Fairly good		594 (15.3)	611 (15.1)
Not so good		268 (6.9)	421 (10.4)

^a^ Categorical variables summarized as *n*(%), continuous variables as mean(SD) or median(25th, 75th centile), see further details in supplementary material.

^b^ Defined according to the Registrar general's classification and included the following categories: professional/managerial, skilled non‐manual, skilled manual, semiskilled/unskilled manual (includes no male head at birth/carer/armed forces/sick/unemployed).

### 2.4 Supplementary analysis

To assess the extent any bias in associations were due to including self‐reported PA estimates in the derivation of NETCRF, we repeated the analysis using a formula for NETCRF which did not include self‐reported estimates of habitual PA^11^ (see details in Supplementary material).

## 3 RESULTS

### 3.1 Sample characteristics

Mean (SD) NETCRF at 45 years was higher in males (11.8 METS [1.2]) than females (9.0 METS [1.3]) (Table 1). All cognitive function task scores at 50 years were slightly higher in females, for example, on the word recall task, females were able to recall, on average, 6.7 (1.5) words compared to 6.4 (1.5) for males.

### 3.2 Association between NETCRF (45 years) and cognitive function (50 years)

In unadjusted analyses, higher NETCRF was associated with higher cognitive function across all measures: for example, a 1‐unit higher NETCRF was associated with a 0.03 (95% CI: 0.01, 0.05) and 0.05 (95% confidence interval [CI]: 0.03, 0.06) higher composite cognitive function *z*‐score, in males and females, respectively (Table 2). In adjusted analyses, while consistent positive associations were observed across most cognitive function outcomes in both sexes, all confidence intervals included the null: for example, a 1‐unit higher NETCRF was associated with a 0.01 (95% CI: −0.02, 0.03) and 0.02 (95% CI: −0.00, 0.04) higher composite cognitive function *z*‐score, in males and females, respectively.

**TABLE 2:** Association between NETCRF and cognitive outcomes (*n* = 8130)^a^.

Mean difference (95% CI) in cognitive function^b^ outcome z‐scores					
	Immediate verbal memory	Verbal fluency	Visual processing speed	Delayed verbal memory	Overall cognition^d^
Males (*N* = 3992)					
Unadjusted	0.05 (0.02, 0.08)	0.04 (0.01, 0.07)	0.01 (−0.02, 0.04)	0.04 (0.01, 0.06)	0.03 (0.01, 0.05)
Adjusted^c^	0.01 (−0.03, 0.04)	0.02 (−0.01, 0.06)	0.01 (−0.03, 0.04)	−0.00 (−0.04, 0.04)	0.01 (−0.02, 0.03)
Females (*N* = 4138)					
Unadjusted	0.04 (0.01, 0.06)	0.06 (0.04, 0.09)	0.05 (0.02, 0.07)	0.04 (0.01, 0.06)	0.05 (0.03, 0.06)
Adjusted^c^	−0.01 (−0.04, 0.03)	0.04 (0.00, 0.07)	0.03 (−0.01, 0.07)	0.02 (−0.02, 0.05)	0.02 (−0.00, 0.04)

^**a**^ Based on 35 imputed datasets.

^b^ Immediate verbal memory = number words recalled in 2 min; Verbal fluency = number of animals named in 1 minute; Visual processing speed = total number of words scanned; Delayed memory = number of words (from first memory task) recalled in 2 min, after delay (all modeled as a z‐score for comparability).

^c^ Adjusted for social class (birth), childhood cognitive function (11 years), educational attainment (33 years), physical activity level (42 years), smoking status (42 years), alcohol consumption (42 years), BMI (42 years) and self‐rated health over the previous 12 months (42 years).

^d^ Average of the sum of the four cognitive function outcome z‐scores.

### 3.3 Supplementary analyses

When analyses were rerun using a NETCRF formula which excluded self‐reported estimates of PA, results were in‐line with those reported above (Table S1).

## 4 DISCUSSION

### 4.1 Summary of findings

Using data from a large general population sample followed from birth for over five decades, we examined the relationship between NETCRF in mid‐life and cognitive function 5 years later. While higher NETCRF was associated with better cognitive outcomes in unadjusted analyses, after adjusting for putative confounders, associations between NETCRF and cognitive outcomes largely disappeared.

### 4.2 Interpretation of findings

Our findings are consistent with previous longitudinal studies which observed, in unadjusted analyses, associations between greater baseline CRF and higher scores on similar cognitive function tasks at follow‐up (6–25 years later). Similar to our observations, associations subsequently weakened or disappeared, after adjusting for confounders, though associations with indicators of executive function (e.g., Stroop test) persisted.^12^ Pentikainen and colleagues^13^ also found persistent associations between greater baseline CRF and higher executive function, processing speed and total cognition at 2 years of follow‐up. However, similar to our findings, they did not observe an association between CRF and memory. Given the persistent and specific associations with executive function, it has been speculated that aerobic conditioning exercise (and therefore CRF) may have a specific link with attention/executive function but not with other cognitive function domains.^12^ Inter‐study differences in findings could also be due to differences in the measurement of CRF (non‐exercise testing vs exercise‐testing), life‐stage of participants (45 years vs. 69 years), length of follow up (5 years vs. 2 years) and/or covariates adjusted for. Evidence from trial data is also inconsistent, with two large Cochrane reviews provide conflicting conclusions regarding the efficacy of CRF for improving cognitive function in later life.^14^, ^15^ Future studies should triangulate evidence using different methodological approaches (e.g., longitudinal studies, trial evidence, genetic studies) and a range of tasks assessing different aspects of cognitive function in order to obtain robust and comprehensive evidence regarding the causal relationship between CRF and cognitive function.

### 4.3 Strengths and limitations

Our study has a number of strengths including its large prospective design with multiple follow‐up time points which enabled us to respect the temporal ordering of our exposure, outcome and confounding variables. This reduces the possibility of reverse causation. The use of an age‐homogenous sample means cognitive function was assessed at approximately the same age (50 years) for all individuals, thus removing the known influence of age on cognition. Although we accounted for cognitive function in childhood and educational attainment in adulthood, measures of cognition in adulthood (e.g., at 45 years) were not available. This lack of repeat outcome data using consistent measurements in mid‐life meant we were unable to interrogate the association between NETCRF at 45 years and cognitive function at 50 years more thoroughly. Specifically, observed associations could reflect an association between NETCRF and cognitive function in earlier adulthood, which subsequently tracks over time. Relatedly, the lack of required data to derive NETCRF at 50 years means it was not possible to examine change in NETCRF and cognitive function at 50 years. The NETCRF equation used in this study has been well validated in adults^16^ and is an appropriate proxy for CRF in large scale studies, such as the 1958 birth cohort. Nonetheless, we acknowledge that NETCRF is not equivalent to the gold‐standard assessment of CRF via tests to exhaustion measuring oxygen uptake.^3^ While availability of detailed, prospectively collected covariate data enabled us to account for several important baseline confounders, the possibility of residual confounding cannot be ruled out. As in all longitudinal studies, loss to follow‐up has occurred. While participants in mid‐adulthood were broadly representative of the original population, the most disadvantaged participants were least likely to remain.^7^ Furthermore, our average NETCRF estimates (males: 11.8 METS; females: 9.0 METS) indicate a relatively fit sample when compared to adults of a similar age in the general population.^17^ The possibility of selection bias should therefore be acknowledged. Finally, as the 1958 birth cohort is predominantly of White British ethnicity (approximately 98% at 45 years); we are unable to generalize results to other ethnic groups.

## 5 CONCLUSION

Higher NETCRF at 45 years was associated with better cognitive function at 50 years, however, associations disappeared when adjusted for sociodemographic and behavioral factors. Further research triangulating evidence obtained from more detailed longitudinal studies (i.e., with repeat measurements of CRF and cognition), different data sources (e.g., genetic and brain imaging studies) and analytical methods (e.g., life course approach, Mendelian randomization), is required to establish the causal relationship between CRF and cognitive function.

## FUNDING INFORMATION

This work was funded by a UK Medical Research Council Career Development Award (ref: MR/P020372/1) awarded to SMPP. MH/JB are supported by a British Heart Foundation grant (SP/F/20/150002). JJM is funded by an MRC grant (MR/N013867/1). The funders had no role in study design, data collection and analysis, decision to publish, or preparation of the manuscript.

## CONFLICT OF INTEREST STATEMENT

The authors have declared that no competing interests exist.

## Supporting information


Data S1:

## Data Availability

The original data for the 1958 NCDS are available from the UK Data Service; applications for access to any data held by the UK Data Archive that forms part of the NCDS Biomedical Resource will require special license and should be submitted to clsfeedback@ioe.ac.uk.
