# Supplementary material for: Is cardiorespiratory fitness associated with cognitive outcomes in mid‐adulthood? Findings from the 1958 British birth cohort
Source: Scand J Med Sci Sports. 2023 Oct 18;33(12):2613–9. doi: 10.1111/sms.14525 (PMC10946453; doi:10.1111/sms.14525)
Supplement: Supplementary file 1 — Data S1: [file SMS-33-2613-s001.docx]

**Supplementary material: Is cardiorespiratory fitness associated with cognitive outcomes in mid-adulthood? Findings from the 1958 British birth cohort**

Pinto Pereira, S.M.,^1^ Mitchell, J.J.,^1^ Blodgett, J.M.,^1^ Hamer, M.^1^ & Norris, T.^1^*

^1^Institute of Sport, Exercise and Health, Division of Surgery and Interventional Science, Faculty of Medical Sciences, UCL, London, UK

*Corresponding author:

Tom Norris

Institute of Sport, Exercise and Health, Division of Surgery and Interventional Science, Faculty of Medical Sciences, UCL, London, UK

Email: [t.norris@ucl.ac.uk](mailto:t.norris@ucl.ac.uk)

**Contents**

[**Variable derivation** 2](#_Toc134534711)

[**Supplementary figure S1: Sample flow diagram** 3](#_Toc134534712)

[**Supplementary figure S2. Directed acyclic graph*** 4](#_Toc134534713)

[**Supplementary table 1 Association between NETCRF* and cognitive outcomes (n=8,130)**** 5](#_Toc134534714)

# **Variable derivation**

*Covariates*

Social class at birth (or at 7y if missing at birth) was defined according to the Registrar general’s classification and included the following categories: professional/managerial, skilled non-manual, skilled manual, semiskilled/unskilled manual (includes no male head at birth/carer/armed forces/sick/unemployed). Cognitive function in childhood was calculated as the mean score obtained on a reading comprehension and maths test administered by schoolteachers at 11y. The reading comprehension test consisted of 35 sentences (1 mark per sentence) in which the child was required to read a sentence and choose from a selection of 5 words the most appropriate to complete the sentence. From the list, the child was required to underline the missing item which completed the sentence. The maths test consisted of 40 items (1 mark per item). The test included number skills, fractions, measures and geometry. Most questions were calculated directly, with a few involving multiple-choice answers. Further details of these assessments can be found on the CLOSER website(1). Educational attainment was defined as the highest academic qualification obtained by age 33y. Physical activity frequency (42y) was categorised into four groups: 4-7 times per week, 2-3 times per week, once per week, ≤3 times per month. Smoking status (42y) was dichotomised into: never/ex- vs current smoker. Alcohol consumption frequency (42y) was categorised into four groups: never, rarely, 2/3/4 times per month, at least twice per week. Self-reported height and weight were collected at 42y and BMI (kg/m^2^) calculated. Self-rated health over the previous 12 months (42y) was categorised as: excellent, good, fairly good, not so good.

*Non-exercise testing cardiorespiratory fitness (NETCRF, 45y) derived without self-reported physical activity*

We used the formula from de Souza at al(2), which does not include physical activity data:

NETCRF (ml/kg/min) = 45.2 - 0.35(age) - 10.9(sex) - 0.15(weight [lbs]) + 0.68(height [in]) - 0.46

Estimates of average NETCRF (METS) using this formula were 10.6 (vs 11.8) and 7.8 (vs 9.0), in males and females respectively. There was good agreement between the estimates of NETCRF derived using this formula and that of Stamatakis et al (2013)(3), with a correlation coefficient of 0.89.

1. CLOSER. Cognitive measures in the 1958 National Child Development Study 2022 [Available from: <https://closer.ac.uk/cross-study-data-guides/cognitive-measures-guide/ncds-cognition/>.

2. de Souza e Silva CG, Kaminsky LA, Arena R, Christle JW, Araújo CGS, Lima RM, et al. A reference equation for maximal aerobic power for treadmill and cycle ergometer exercise testing: Analysis from the FRIEND registry. European Journal of Preventive Cardiology. 2018;25(7):742-50.

3. Stamatakis E, Hamer M, O'Donovan G, Batty GD, Kivimaki M. A non-exercise testing method for estimating cardiorespiratory fitness: associations with all-cause and cardiovascular mortality in a pooled analysis of eight population-based cohorts. European heart journal. 2013;34(10):750-8.

# **Supplementary figure S1: Sample flow diagram**


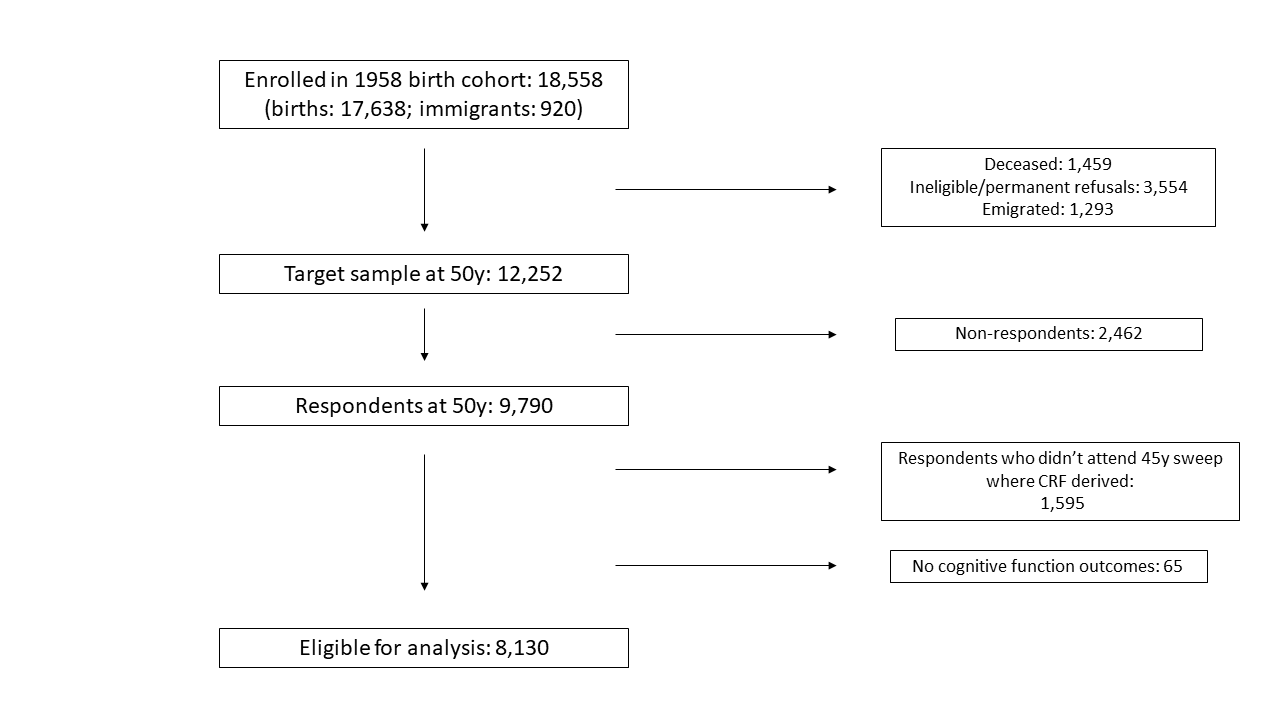


# **Supplementary figure S2. Directed acyclic graph***


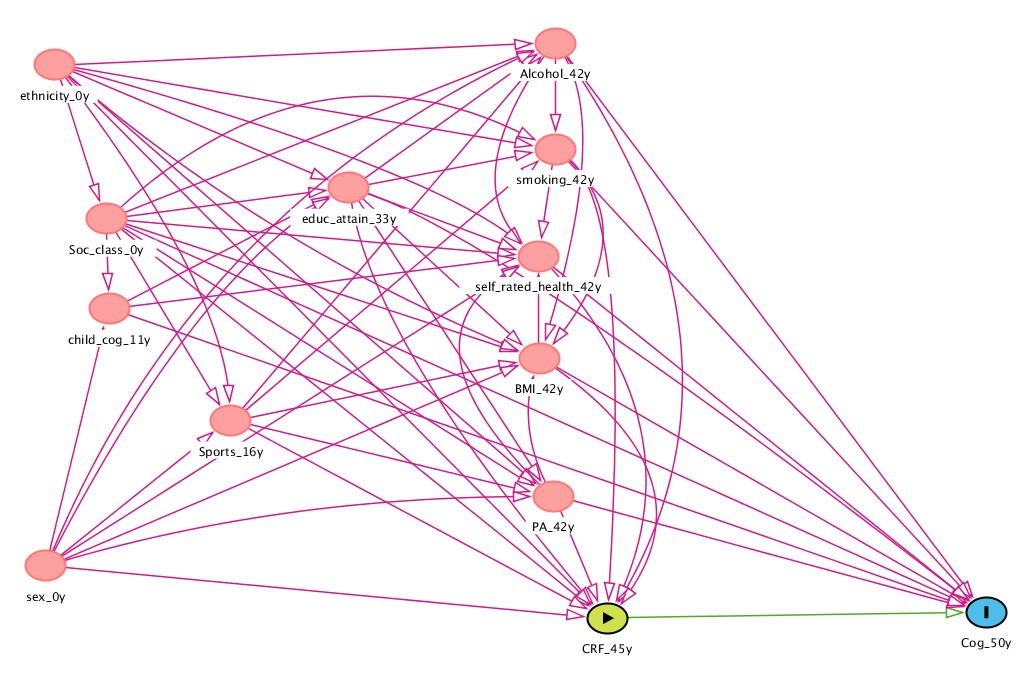


*Based on the above DAG, the minimum adjustment set identified included: social class at birth (*soc_class_0y*), childhood cognitive function (11y) (*child_cog_11y*), educational attainment (33y) (*educ_attain_33y*), physical activity level (42y) (*PA_42y*), smoking status (42y) (s*moking_42y*), alcohol consumption (42y) (*Alcohol_42y*), BMI (42y) (*BMI_42y*) and self-rated health in previous 12 months (42y) (*self_rated_health_42y*).

# **Supplementary table 1 Association between NETCRF^a^ and cognitive outcomes (n=8,130)^b^**

| **Mean difference (95% CI) in cognitive function^c^ outcome z-scores** | | | | | |
| --- | --- | --- | --- | --- | --- |
|  | Immediate verbal memory | Verbal fluency | Visual processing speed | Delayed verbal memory | Overall cognition^e^ |
| Males (N=3,992) | | | | | |
| Unadjusted | 0.01 (-0.01, 0.01) | 0.00 (-0.00, 0.01) | -0.01 (-0.02, 0.01) | 0.01 (-0.00, 0.01) | 0.00 (-0.00, 0.01) |
| Adjusted^d^ | -0.01 (-0.03, 0.00) | -0.01 (-0.02, 0.01) | 0.01 (-0.03, 0.05) | -0.00 (-0.02, 0.01) | -0.01 (-0.02, 0.00) |
| Females (N=4,138) | | | | | |
| Unadjusted | 0.01 (0.00, 0.02) | 0.01 (0.01, 0.02) | 0.01 (0.00, 0.01) | 0.01 (0.00, 0.01) | 0.01 (0.01, 0.01) |
| Adjusted^d^ | -0.01 (-0.03, 0.00) | -0.00 (-0.02, 0.01) | -0.00 (-0.02, 0.01) | 0.00 (-0.01, 0.01) | -0.00 (-0.01, 0.00) |

**^a^**NETCRF derived using formula from de Souza et al. 2018;25(7):742-750; **^b^**based on 35 imputed datasets; ^c^immediate verbal memory= number words recalled in 2 minutes; Verbal fluency= number of animals named in 1 minute; Visual processing speed= total number of words scanned; Delayed memory= number of words (from first memory task) recalled in 2 minutes, after delay (all modelled as a z-score for comparability); ^d^adjusted for social class (birth), childhood cognitive function (11y), educational attainment (33y), physical activity level (42y), smoking status (42y), alcohol consumption (42y) and BMI (42y); ^e^average of the sum of the four cognitive function outcome z-scores
